# An Exploratory Study of the Relationships Between Diesel Engine Exhaust Particle Inhalation, Pulmonary Inflammation and Anxious Behavior

**DOI:** 10.3390/ijerph18031166

**Published:** 2021-01-28

**Authors:** Sunyoung Jeong, Jong-Hwa Lee, Jung-Heun Ha, Jinhee Kim, Inyong Kim, Sungryong Bae

**Affiliations:** 1 Department of Human and Environmental Toxicology, University of Science and Technology, Daejeon 34114, Korea; sunyoung.jeong@kitox.re.kr; 2 Bioanalytical and Pharmacokinetic Research Group, Korea Institute of Toxicology, Daejeon 34114, Korea; jhl@kitox.re.kr; 3 Research Center for Industrialization of Natural Neutralization, Dankook University, Cheonan 31116, Korea; ha@dankook.ac.kr (J.-H.H.); ikim@dankook.ac.kr (I.K.); 4 Department of Food Science and Nutrition, Dankook University, Cheonan 31116, Korea; 5 Drug Information Platform Center, Korea Research Institute of Chemical Technology, Daejeon 34114, Korea; jhkim2@krict.re.kr; 6 Department of Fire Protection and Disaster Management, Chosun University, Gwangju 61452, Korea

**Keywords:** diesel engine exhaust particles, soot, pulmonary inflammation, open field test, anxiety

## Abstract

Recent technical developments brought negative side effects such as air pollution and large-scale fires, increasingly exposing people to diesel engine exhaust particles (DEP). Testing how DEP inhalation triggers pathophysiology in animal models could be useful in determining how it affects humans. To this end, the aim of this study was to investigate the effects of pulmonary exposure to DEP for seven consecutive days in experimental male C5BL6/N mice. Twenty-four C5BL6/N mice were treated with one of the three test materials: distilled water for control, a low DEP exposure (5 mg/kg), or a high DEP exposure (15 mg/kg). Exposure to DEP induced decreased body weight; however, it gradually increased pulmonary weight in a DEP-dose-dependent manner. DEP exposure significantly elevated soot accumulation in the lungs, with the alteration of pulmonary homeostasis. It also elevated infiltrated immune cells, thus significantly increasing inflammatory cytokine mRNA and protein production in the lungs and broncho-alveolar lavage fluid, respectively. Pulmonary DEP exposure also altered behavioral responses in the open field test (OFT). Low exposure elevated moving distance and speed, while significantly decreasing the number of trials to enter the central zone. Different concentrations of DEP resulted in different behavioral changes; however, while anxiety levels increased, their degree was independent of DEP concentrations. Results suggest that DEP exposure may possess pro-inflammatory responses in the lungs and trigger anxiety.

## 1. Introduction

Diesel engine exhaust particles (DEP) are the gaseous end-product of organic materials after combustion with particulates. DEP are a complex aerosol particulate matter, and their composition is atypical since they are generated from various internal combustion engines that use different fuel types, injection rates, and operational conditions [1]. DEP are the pyrosynthetic products of petroleum fuel, including nitrogen (N_2_), oxygen (O_2_), carbon dioxide (CO_2_), water (H_2_O), carbon monoxide (CO), nitrogen oxides (NO_x_), hydrocarbons (HC), aldehyde, and particulate matter [2,3,4,5,6,7,8]. Moreover, the DEP contain traceable and remarkably carcinogenic chemicals, classified by the International Agency for Research on Cancer (IARC) as group 1 (arsenic, benzene, cadmium, formaldehyde, and benzo(a)pyrene), group 2A (1,3-butadiene), group 2B (acetaldehyde, naphthalene, nickel, and styrene) carcinogens, and group 3 of possible carcinogens (acrolein, aniline, fluoranthene, toluene, and xylene) [9]. These carcinogenic DEP may be delivered to pulmonary systems through inhalation, as a type of dispersible soot and/or fine particulate matter, which may trigger carcinogenesis in humans [10].

Following the Industrial Revolution, we have been experiencing highly advanced and organized technology in our lives. However, technical development has inevitably brought negative side effects, such as air pollution and large-scale fires in both metropolitan and wild areas. Air pollution and large fires contain numerous combustion products, and DEP are one of such significant mixtures of combustion products. Inhalation and accumulation of soot and DEP-filled particulate matter to the pulmonary and circulatory systems may cause pathophysiological adverse effects. Exposure to DEP may elevate the risk of the onset of carcinogenesis [10], lung and heart impairments [11], and mental disorders [12].

With the increasing chances of DEP exposure through air pollution and large fires, we need a robust in vivo model to test how DEP inhalation triggers pathophysiology in animal models to extrapolate the results to humans. However, DEP inhalation and its pathophysiological understanding are at an incipient phase; therefore, we may have to develop and update pre-existing experimental DEP inhalation models. To date, DEP inhalation models vary in frequency and dosage of DEP exposure methods. Among various reports, one crucial conclusion is the elevation of inflammatory mRNA and/or protein expression in DEP-exposed rodents [13,14,15,16,17]. However, these reports were inconclusive regarding the dose-dependent effects of DEP, inflammation, pulmonary oxidative stress, and behavioral alterations.

In abrupt and rapid pulmonary DEP exposure of evacuees and/or firefighters, the accumulation of soot in lung cells may increase, with an elevation of inflammatory and carcinogenic responses [18,19]. DEP exposure may affect not only local pulmonary pathophysiology but also behavioral responses. Intact behavioral responses are highly important in fires, since the limitation of movement of evacuees and/or firefighters is directly related to the number of causalities [20]. Therefore, understanding the behavioral changes in DEP exposure in the preclinical experimental model is highly necessary.

In this study, we attempted to understand inflammatory responses in the lungs and behavioral changes in young adult male mice in response to DEP exposure in a dose-dependent manner (0, 5, and 15 mg/kg). We verified the DEP-inducible pulmonary inflammatory murine model and investigated pathophysiological effects based on histological images and protein and mRNA expressions. In addition, we carefully observed behavioral changes caused by DEP exposure in the experimental mouse model.

## 2. Materials and Methods 

### 2.1. Animal Treatments

In vivo experiments were approved by the Institutional Animal Care and Use Committee of the Korea Institute of Toxicology. Seven-week-old, male, C57BL/6NCrlOri mice were purchased from Orient Bio, Inc. (Seongnam-Si, Korea) and were housed in polycarbonate cages (135W × 280L × 145H mm) in a room with controlled temperature (22 ± 3 °C), humidity (50 ± 20%), and a 12-h light/dark cycle. The experimental mice (*n* = 24) were provided with a sterilized commercial pellet diet (PMI Nutrition International, St. Louis, MO, USA) and filtered water ad libitum. After 5 days of acclimation, they were randomly assigned into three groups (*n* = 8 per group). Commercially available DEP (SRM 2975; National Institute of Standards and Technology, Gaithersburg, MD, USA) were dispersed in 50 µL distilled water and were administered in a low (5 mg/kg; *n* = 8) and a high (15 mg/kg; *n* = 8) dosage (thus creating the low DEP exposure (DEPL) and high DEP exposure (DEPH) groups) by intratracheal instillation for 7 consecutive days. Low dosage of DEP was selected based on the previous report that 100 μg/head (≈50 mg/kg) of DEP (SRM 2975) inhalation caused significant pulmonary inflammation [13]. The highest dosage (15 mg/kg) was set 3 times higher concentration of low dosage. The control (Ctrl; *n* = 8) group was treated with distilled water for 7 days. The experimental mice were weighed and sacrificed on the 8th day. Collected tissues were weighed, snap-frozen, and stored at −80 °C for further analysis.

### 2.2. Histological Analysis

After 24 h from the last DEP instillation, the experimental mice were weighed and sacrificed by isoflurane (Sigma-Aldrich, St Louis, MO, USA) for histological examination. The left lung tissue was removed and fixed in 10% (*v*/*v*) neutral-buffered formalin for 24 h and then embedded in paraffin. Pulmonary tissue sections (4 µm thickness) were deparaffinized with xylene (Sigma-Aldrich, St Louis, MO, USA) and histochemically stained with hematoxylin (Sigma-Aldrich, St Louis, MO, USA) and eosin (Sigma-Aldrich, St Louis, MO, USA) [21,22]. The stained sections were analyzed under a light microscope (Carl Zeiss, Oberkochen, Germany).

### 2.3. Collection of BALF (Broncho-Alveolar Lavage Fluid)

Broncho-alveolar Lavage Fluid (BALF) was collected 24 h after the last DEP instillation. After the mice were anesthetized with isoflurane (Sigma-Aldrich, St Louis, MO, USA), 0.7 mL PBS (Sigma-Aldrich, St Louis, MO, USA) per head was instilled into the right lungs via the tracheal tube. The collected BALF was centrifuged (2000 rcf for 5 min at 4 °C). After centrifugation, the supernatant was stored at −80 °C for cytokine analysis. A portion of the remaining cell pellets was resuspended in 100 µL PBS, and the rest was made into cell smears. The total number of cells was counted using a NucleoCounter (NC-250; ChemoMetec, Gydevang, Denmark). For counts of different cell types, BALF cell smears were prepared using Cytospin (Thermo Fisher Scientific, Waltham, MA, USA) and stained with Diff-Quik solution (Dade Diagnostics, Aguada, Puerto Rico). The distributions of the alveolar macrophages, eosinophils, neutrophils, and lymphocytes were assessed based on their characteristic cell shapes. A total of 300 cells per slide were counted.

### 2.4. Measurements of H_2_O_2_ and Cytokines

The measurements of cytokine levels of hydrogen peroxide, TNFα, and interleukin-6 (IL-6) levels in BALF were quantified by ELISA, using commercial kits (Thermo Fisher Scientific, Waltham, MA, USA) according to the manufacturer’s protocol.

### 2.5. Quantitative RT-PCR Analysis

A quantitative RT-PCR (qRT-PCR) was performed to assess TNFα, IL-1β, and IL-6 mRNA levels in DEPL- and DEPH-treated lungs and livers of mice. The total RNA was isolated using the RNeasy Mini kit (Qiagen, Germantown, MD, USA) according to the manufacturer’s instructions. Extracted RNA was transcribed to cDNA using primer sequences. Real-time PCR was performed using a SYBR Green PCR Master Mix (Applied Biosystems, Waltham, MA, USA) according to the manufacturer’s protocol [21,22]. Samples from 8 animals per treatment group were analyzed and presented relative to GAPDH mRNA expression. The oligonucleotide primers used in this study are summarized in Table 1.

### 2.6. Open Field Test

At one hour after the last DEP instillation, an open field test was performed. Mice were individually placed in the center of a square Plexiglass container with a field measuring 42 cm × 42 cm × 42 cm. The apparatus was illuminated by a 100 W lamp placed 2 m above the center of the floor of the apparatus. Mice were allowed to freely explore the area and were given a 10 min habituation period followed by 10 min of behavioral recordings. A computer system (Ethovision, Noldus, Netherlands) recorded and calculated the movement distance (in), duration (sec), and velocity (in/sec) of each subject.

### 2.7. Statistical Analysis

Data are presented as mean ± standard mean error. The results were analyzed by one-way analysis of variance (ANOVA; GraphPad PRISM 8, San Diego, CA, USA) followed by Tukey’s post-hoc test to examine the impact of DEP inhalation at a low or a high levels of DEP exposure compared to a control group. Statistical significance was considered at *p* < 0.05.

## 3. Results

### 3.1. Physiological Changes of Body and Organ Weights

**Figure 1:**
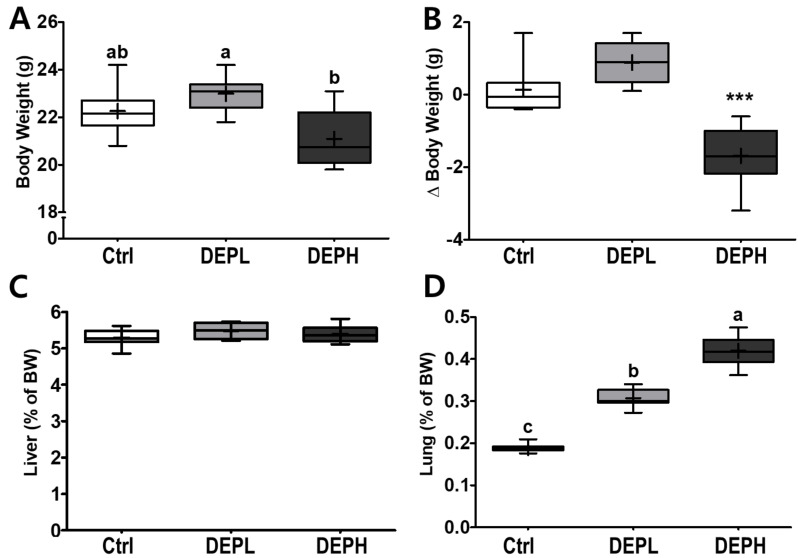
Box-and-whisker plots representing physiological changes in diesel exhaust particles (DEP) exposed mice on (**A**) body weight (BW); (**B**) change of BW (final BW minus initial BW); (**C**) liver weight/BW ratio; and (**D**) lung weight/BW ratio; *n* = 8 for each group. A one-way ANOVA followed by Tukey’s post-hoc test was performed; labeled means without a common letter differ (*p* < 0.05); *** *p* < 0.001.

DEP inhalation did not have a significant effect on final body weights at a low dose (DEPL; Figure 1A), whereas DEPH mice had a significant decrease in the delta body weights (the final BW minus the initial BW) compared to either the Ctrl or DEPL groups (Figure 1B). No remarkable differences were observed in the relative weights of the liver (Figure 1C). However, the relative weights of the lungs gradually increased in a dose-dependent manner (Figure 1D) in DEP-exposed mice. Therefore, the DEPH and DEPL groups had the highest and second highest lung weights, respectively.

### 3.2. Histological Analysis

**Figure 2:**
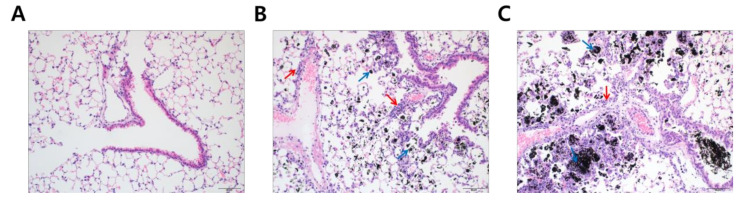
Soot accumulation in the experimental lung after diesel exhaust particles (DEP) exposure. Lung tissues were microscopically evaluated with hematoxylin and eosin staining (20×). The panels represent mice treated with (**A**) distilled water; (**B**) 5 mg/kg of DEP (DEPL); and (**C**) 15 mg/kg of DEP (DEPH) for 7 consecutive days. Blue arrows in (**B**,**C**) point to black-pigment-laden macrophages in the alveoli and alveolar lumen interstitium. Red arrows in (**B**,**C**) indicate the infiltrated inflammatory cell in peribronchiolar, perivascular, and interstitial regions.

To understand why DEP-exposed mice had increased lung weights, hematoxylin and eosin (H&E) staining was performed to analyze histological changes in lung tissue following DEP-inhalation. DEP exposure resulted in a significant accumulation of black particle-laden alveolar macrophages and soot-like black particles in the alveolar lumen in a dose-dependent fashion (Figure 2, blue arrows). Moreover, inflammatory cells infiltrated the peribronchiolar, perivascular, and interstitial regions (Figure 2, red arrows). Based on the histological analysis, we may assume that DEP deposition in the lung was the primary reason for increased lung weights in the experimental mice.

### 3.3. Cell Contents in Bronchial Alveolar Lavage Fluids (BALF)

**Figure 3:**
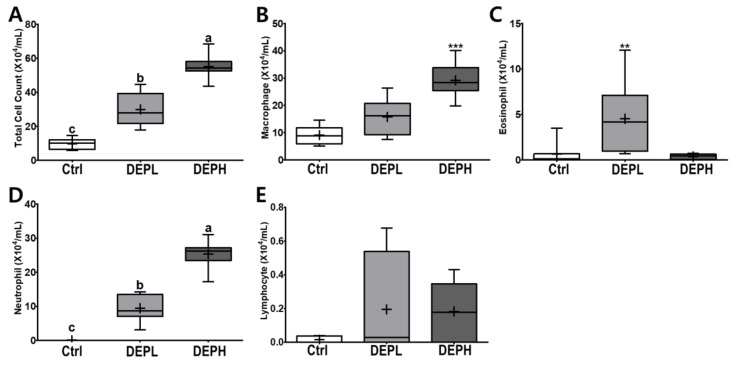
Box-and-whisker plots representing the absolute number of inflammatory cell profiles in the broncho-alveolar lavage fluid (BALF) after repeated exposures to diesel exhaust particles (DEP) for 7 consecutive days. All the cells within the BALF were counted and differentiated: (**A**) the total number of cells, (**B**) macrophages, (**C**) eosinophils, (**D**) neutrophils, and (**E**) lymphocytes; *n* = 8 for each group. One-way ANOVA was performed, followed by Tukey’s post-hoc test; labeled means without a common letter differ (*p* < 0.05); ** *p* < 0.01; *** *p* < 0.001.

**Figure 4:**
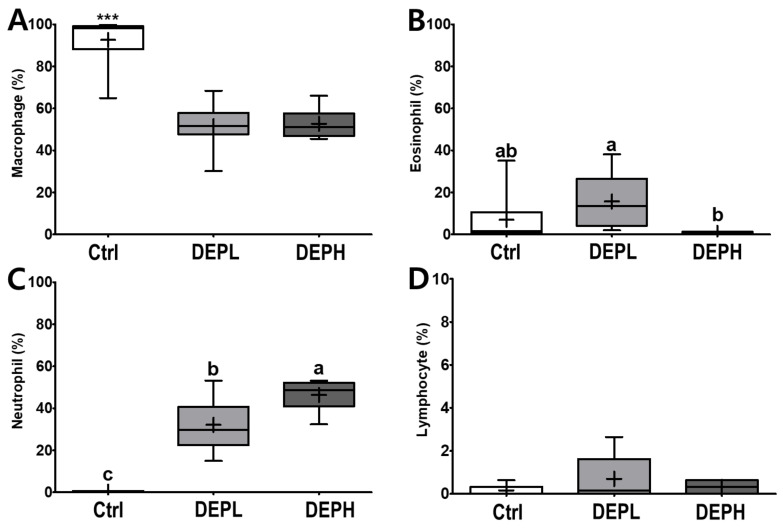
Box-and-whisker plots representing the relative ratio of inflammatory cell profiles in the broncho-alveolar lavage fluid (BALF) after repeated exposures to diesel exhaust particles (DEP) for 7 consecutive days. The (**A**) macrophages, (**B**) eosinophils, (**C**) neutrophils, and (**D**) lymphocytes are expressed as percentages of the total number of cells; *n* = 8 for each group. One-way ANOVA was performed, followed by Tukey’s post-hoc test; labeled means without a common letter differ (*p* < 0.05); *** *p* < 0.001.

DEP exposure is closely related to pulmonary inflammation. To detect inflammatory responses in the lungs, the total number of cells in the BALF was measured after the DEP instillation (Figure 3A). The absolute number of inflammatory cells, including macrophages and neutrophils, was increased compared to that of the control group (Figure 3B,D). However, the relative ratio of macrophages per total cells in DEP-treated mice was lower compared to that of the control group, regardless of DEP concentration (Figure 4A). By contrast, DEP induced a relative ratio of neutrophils in the total number of dosed cells (Figure 4C). The number of eosinophils was slightly higher in the DEPL group; however, the ratio of eosinophils to the total number of cells showed no statistical change and recovered to the normal level in the DEPH group (Figure 3C and Figure 4B). DEP instillation had no significant effect on lymphocytes within BALF (Figure 3E and Figure 4D).

### 3.4. Pro-Inflammatory Cytokines and H_2_O_2_ in BALF and Liver

**Figure 5:**
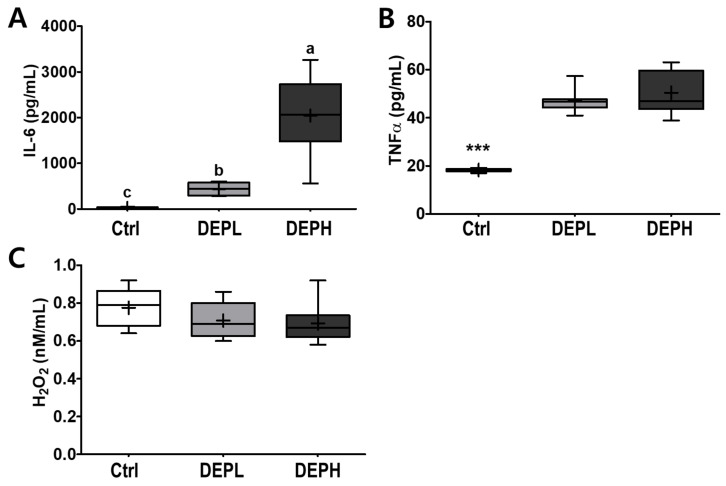
Box-and-whisker plots showing interleukin 6 (IL-6), tumor necrosis factor-alpha (TNFα), and hydrogen peroxide (H_2_O_2_) levels in broncho-alveolar lavage fluid (BALF) after repeated exposures to diesel exhaust particles (DEP) for 7 consecutive days: (**A**) IL-6, (**B**) TNFα, and (**C**) H_2_O_2_; *n* = 8 for each group. A one-way ANOVA followed by Tukey’s post-hoc test was performed; labeled means without a common letter differ (*p* < 0.05); *** *p* < 0.001.

**Figure 6:**
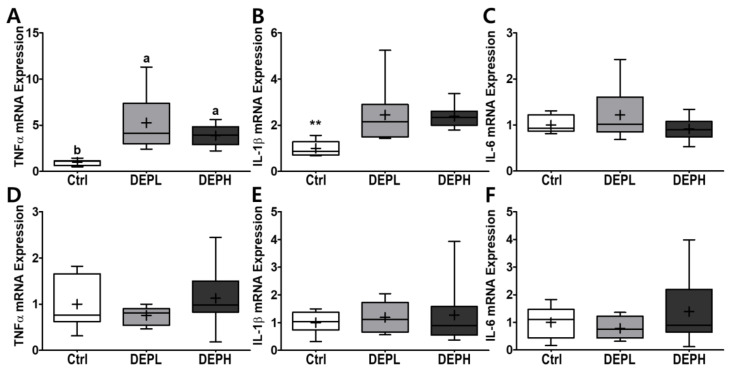
Box-and-whisker plots representing pulmonary and hepatic inflammatory cytokine mRNA expressions after diesel exhaust particles (DEP) exposure. Pulmonary (**A**) tumor necrosis factor-alpha (TNFα), (**B**) interleukin 1 beta (IL-1β), and (**C**) IL-6, as well as hepatic (**D**) TNFα, (**E**) IL-1β, and (**F**) IL-6, were analyzed through qRT-PCR by normalization of glyceraldehyde-3-phosphate dehydrogenase (GAPDH) mRNA expression; *n* = 8 for each group. A One-way ANOVA followed by Tukey’s post-hoc test was conducted; labeled means without a common letter differ (*p* < 0.05); ** *p* < 0.01.

To verify the elevated inflammatory responses in the lungs caused by DEP exposure, we measured the cytokine protein levels (IL-6 and TNFα) within BALF. The levels of IL-6 in BALF were elevated in a DEP-dose-dependent manner. The DEPL and DEPH groups had elevated IL-6 protein levels 16.8- and 79.3-fold higher than the BALF of the control group (Figure 5A). Moreover, TNFα was also significantly secreted within DEP-exposed mice; the DEPL and DEPH mice showed an increased TNFα secretion of 2.6- and 2.8-fold, respectively (*p* < 0.001; Figure 5B). In general, chronic inflammation triggers cellular oxidative stress; therefore, we also measured the level of hydrogen peroxide from BALF after DEP exposure. However, in our experimental setting, hydrogen peroxide levels in the BALF did not respond to DEP exposure (Figure 5C). To understand whether pulmonary inflammation by DEP exposure is regulated in a transcriptional manner, mRNA expression of pro-inflammatory cytokines (TNFα, IL-1β, and IL-6) was determined by qRT-PCR. Pulmonary TNFα (*p* < 0.05; Figure 6A) and IL-1β (*p* < 0.01; Figure 6B) mRNA expressions were remarkably elevated in the DEP-exposed groups. However, the IL-6 mRNA expression in the lung was not altered by DEP exposure (Figure 6C). DEP increased pulmonary inflammation at transcriptional and translational levels; therefore, we raised an extended research question of whether DEP exposure may elevate whole-body inflammation via circulation. To answer it, the hepatic pro-inflammatory cytokine mRNA expression was assessed by qRT-PCR. DEP exposure to experimental mice did not alter hepatic TNFα (Figure 6D), IL-1β (Figure 6E), and IL-6 (Figure 6F) mRNA expressions. Moreover, the enzymatic activities of serum alkaline phosphatase, alanine aminotransferase, and aspartate aminotransferase were not different among the experimental groups (data not shown). Therefore, DEP exposure may not trigger systemic inflammation but may induce a local pulmonary one.

### 3.5. Open Field Test (Distance and Time)

**Figure 7:**
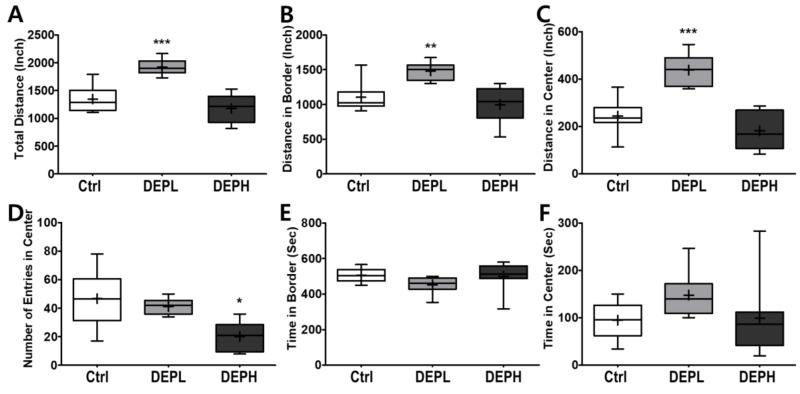
Box-and-whisker plots presenting behavior changes in the open-field after diesel exhaust particles (DEP) exposure. (**A**) The total distance the mice traveled, (**B**) distance traveled in the border area, (**C**) distance traveled in the central area, (**D**) number of entries into the center, (**E**) time spent in the border area, and (**F**) time spent in the center area during the 10 min of the open field test; *n* = 8 for each group. The one-way ANOVA was followed by Tukey’s post-hoc test; * *p* < 0.05; ** *p* < 0.01; *** *p* < 0.001.

Given the finding that DEP exposure induced pulmonary inflammation in a dose-dependent manner, we next examined the effects of DEP exposure on open-field behavior in mice. To evaluate the effect of DEP on murine behavior, the locomotor activity was analyzed using an open field test. A low exposure significantly increased the total distance travelled in the open field (Figure 7A), both along the border (Figure 7B) and in the center (Figure 7C). The high exposure significantly decreased the number of entries to the center compared to the control or DEPL groups (Figure 7D). Alteration of the number of entries to the center by DEP exposure may imply that the anxiety-related behavior increased in a DEP-dose-dependent manner. The amount of time spent at the border (Figure 7E) and in the center (Figure 7F) did not change statistically.

### 3.6. Open Field Test (Velocity)

**Figure 8:**
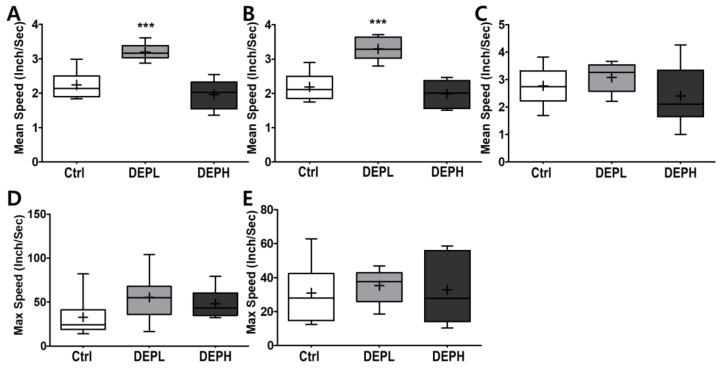
Box-and-whisker plots showing behavior changes in the open-field after diesel exhaust particles (DEP) exposure. (**A**) The total mean speed, (**B**) mean speed on the border, (**C**) mean speed in the center, (**D**) max speed on the border, and (**E**) max speed in the center area during the 10 min of the open field test; *n* = 8 for each group. The one-way ANOVA was followed by Tukey’s post-hoc test; *** *p* < 0.001.

As shown in Figure 7, DEP exposure altered murine behavior; therefore, we logically postulated that DEP exposure would alter moving velocity in the open field test. DEPL exposure induced the average velocity at the border (Figure 8A), which contributed to the total speed (Figure 8B). However, higher exposure to DEP attenuated the moving velocity significantly more than that of the DEPL group (Figure 8A,B). In addition, DEP exposure did not alter the maximum speed at the border (Figure 8D) and in the center (Figure 8E).

## 4. Discussion

The main goal of the current study was to assess physiological changes and pulmonary inflammatory responses associated with DEP exposure in mice in a dose-dependent manner. In addition, we planned to assess behavioral changes caused by DEP exposure in a dose-dependent manner, compared to the control group. We found that animals had larger lung weights along with body weight loss following DEP exposure. We also found that DEP instillation was able to induce inflammatory responses in the lungs by elevating pro-inflammatory cytokine secretion and gene expression. However, in our experiment, DEP exposure did not induce whole-body and systemic inflammation. Moreover, DEP exposure altered behavioral changes in experimental mice, which may relate to anxious behavior.

In this study, DEP exposure to experimental mice caused significant weight loss (Figure 1A,B) in DEPH, and lung weights were remarkably increased by increasing the concentration of DEP in a dose-dependent manner (Figure 1D). Another study also reported that DEP exposure to balb/c mice showed elevated lung weight as well [13]. Even with enlarged lungs, high-dose DEP exposure decreased body weight; therefore, we assumed that DEP exposure may cause systemic and whole-body inflammation. However, in this experimental setting, DEP exposure may not have caused whole-body inflammation since liver weight (Figure 1C), hepatic inflammatory mRNA expression (Figure 6D,E), and biochemical markers (data not shown) in the circulation were intact. Therefore, it is inconclusive; however, DEP exposure may cause other types of partial toxicity since the body weight clearly decreased in DEPH mice (Figure 1A,B).

DEP exposure remarkably increased pulmonary soot accumulation (Figure 2B,C) and inflammation (Figure 2, Figure 3, Figure 4, Figure 5 and Figure 6). The experimental rodents showed significantly elevated cell numbers in the lungs, which may be related to infiltrated immune cells, as summarized in Table 2 [13,14,15,16,17]. The distinctive infiltrated cells in the lungs were macrophages and neutrophils (Figure 3B,D and Figure 4A,C). However, the elevation of recruited inflammatory cells may not be sufficient to explain the increased lung weight following DEP exposure (Figure 1D). Soot accumulation may also account for the increased weight (Figure 2). Physical soot accumulation in the lungs may cause pulmonary and/or systemic hypoxia, since the oxygen and carbon dioxide exchange may not be physically available. To understand our scientific assumption, we may monitor the oxygen consumption and perform a hematological assessment related to oxygen utilization in the circulation and in the kidney in the future, which may relate to body weight loss and possible toxic mechanisms generated by DEP exposure in the extended experiments.

Moreover, we also observed how DEP exposure altered behavior responses of the experimental mice. Due to the recent increase in chances of humans suffering from subchronic inhalation of toxicants [23,24] through air pollution and large fires, we planned an OFT to understand how DEP affected anxious behavior. In the OFT, DEPL increased the total traveled distance (Figure 7A), distance traveled along the border (Figure 7B), and distance covered in the center (Figure 7C). Increased behavioral responses may be related to the increase of the total moving speed (Figure 8A) and speed along the border (Figure 8B). The increased total moving speed in DEPL (Figure 8A) may rely on the elevated moving speed at the border (Figure 8B) rather than on the intact moving speed in the center (Figure 8C). Although low exposure could not be conclusively evaluated to increase anxious response, it is postulated that DEPL augmented the speed along the border, where anxiety occurred. However, enigmatically, high exposure did not alter behavior compared to the control group. One significant behavioral difference in DEPH mice was the attenuated trials to the central zone of the open field (Figure 7D).

In this study, mice gradually decreased the number of entries to the central area with the increase in DEP in a dose-dependent manner (Figure 7D). High exposure did not provoke other anxiety-related responses, such as affecting moving distance and speed, except for significantly decreasing the number of trials to move into the central zone. Considering the behavioral alterations, both high- and low-DEP-exposed groups may suffer anxiety to a different degree. In the clinic, anxiety disorders are classified into four levels: mild, moderate, severe, and panic [25]. Therefore, we carefully postulated that the different behavioral responses between DEPL and DEPH are due to the differences in the level of anxiety in experimental mice. To affirm our logical assumption, we searched for the effects of DEP inhalation on behavioral alterations; however, it was hard to find recent publications. Instead of DEP exposure, we broadened the search index to the effects of particulate matter exposure to experimental animals, and these results are summarized in Table 3 [26,27,28]. The authors used different animal strains and exposure methods; however, all results clearly showed that particulate matter exposure to rodents altered behaviors related to anxiety.

Behavioral alteration is crucially important in both long-term and short-term exposure to DEP. With respect to short-term exposure of DEP, we may think about the large fires. The number of high-rise and large buildings is continuously increasing in metropolises to accommodate the population increases in large-scale cities. While these buildings are useful constructions, they have disadvantages in disaster situations, such as fire, due to the extension of the evacuation distance and the complexity of the evacuation route. Based on these disadvantages, heavy casualties may occur. In the long term, lifetime DEP inhalation from air pollution may cause significant pathological alterations, such as asthma [29,30], allergic reactions [31], and cardiac pressures [32]. Moreover, DEP exposure to neuronal cells elevates neuronal oxidative stress and inflammation [33], as well as neurotoxicity [34]. Extended neuronal stress caused by DEP exposure may increase the emergence of Alzheimer’s disease by accelerating plaque formation [35] and increasing cephalic inflammation and oxidative stress [36]. In addition, air pollution increases the on-set possibility of abnormal cognitive and Alzheimer’s disease [37]. Therefore, DEP could be delivered to the neuronal systems and potentially cause abnormal behavior.

The toxic effect of soot is relatively lower than that of CO and HCN gases, such as CO_2_ and O_2_; however, as we examined in this study, soot has negative effects on mammals. Soot particles are inhaled and impact a larger surface area of lung tissue. Soot accumulation in the lungs may disrupt the transfer of oxygen-carbon dioxide to the circulation, causing significant disorders that include acute short-term and chronic long-term pathophysiological symptoms, such as respiratory, cardiopulmonary, and allergic responses. Moreover, as this study showed, pulmonary soot accumulation may increase anxiety-related behaviors. In the future, we may deeply consider assessing anxiety-related hormones and neural responses in the brain.

## Figures and Tables

**Table 1:** qRT-PCR primer sequences (5′ to 3′).

Transcript	Forward Primer	Reverse Primer
TNFα	GGCTGCCCCGACTACGT	ACTTTCTCCTGGTATGAGATAGCAAAT
IL-6	CTGCAAGAGACTTCCATCCAGTT	AGGGAAGGCCGTGGTTGT
IL-1β	GTCACAAGAAACCATGGCACAT	GCCCATCAGAGGCAAGGA
GAPDH	CATGGCCTTCCGTGTTCCTA	GCGGCACGTCAGATCCA

**Table 2:** Selected studies of diesel exhaust particles exposure.

Ref.	Age or BW	Strain	DEP Exposure Time	Biological Markers	Results
[13]	~16.3 g	balb/c mouse	7 days	Lung weight BALF	↑ Cell # ↑ DEP accumulation↑
[14]	6–7 wks	ICR mouse	Once	Histology in lung BALF	Edema Cell # ↑
[15]		C57BL/6 mouse	1, 4, 7 days	BALF	Cell # ↑ Cytokine P ↑
[16]		C57BL/6 mouse	1, 4, 7 days	BALF	Cell # ↑ Cytokine P ↑
[17]	~175 g	SD rat	once and sacrificed after 1, 7 and 30 days	BALF Lung	Cytokine M/P ↑ Cytokine M ↑ LDH activity↑

increased (↑); number (#); protein (P); mRNA (M), lactate dehydrogenase (LDH).

**Table 3:** Selected behavioral studies after particulate matter (PM) exposure by behavior test.

Ref.	Age	Strain	PM Exposure Time	Biological Markers	Results
[26]	4 wks	pregnant ICR mouse	6 h/day, 14 d (early prenatal exposure)	total distance moved duration in peripheral zone duration in central zone frequencies of zone transition velocity of movement duration of movement hyperactivity	↑ ↓ ↑ ↑ ↑ ↑ ↑
[27]	7 wks	NMRI mouse	3, 6, 8 h, 5 d, 12 wks	Forced swimming test time spent in open arms numbers of entries into open arms	immobility↑ ↓ ↓
[28]		SD rat	7 d	Total distance Speed of mobility	↓ ↓

increased (↑); decreased (↓).

## Data Availability

The data presented in this study are available from the corresponding author upon reasonable request.
